# Health care providers’ perceptions of using misoprostol in the treatment of incomplete abortion in Malawi

**DOI:** 10.1186/s12913-022-08878-3

**Published:** 2022-12-03

**Authors:** Cecilie Annette Wagenheim, Hedda Savosnick, Bertha Magreta Chakhame, Elisabeth Darj, Ursula Kalimembe Kafulafula, Alfred Maluwa, Jon Øyvind Odland, Maria Lisa Odland

**Affiliations:** 1 Department of Public Health and Nursing, Norwegian University of Science and Technology, Trondheim, Norway; 2 Kamuzu University of Health Sciences, Blantyre, Malawi; 3 Malawi University of Science and Technology, Blantyre, Malawi; 4 School of Health Systems and Public Health, Faculty of Health Sciences, University of Pretoria, Pretoria, South Africa; 5 Department of Obstetrics and Gynecology, St Olav’s Hospital, Trondheim University Hospital, Trondheim, Norway; 6 Malawi-Liverpool-Wellcome Trust Research Institute, Blantyre, Malawi; 7 Institute of Life Course and Medical Sciences, University of Liverpool, Liverpool, UK

**Keywords:** Incomplete abortion, Misoprostol, Post-abortion care, Unsafe Abortion, Health care providers, Perceptions, Malawi

## Abstract

**Background:**

In Malawi, abortion is only legal to save a pregnant woman’s life. Treatment for complications after unsafe abortions has a massive impact on the already impoverished health care system. Even though manual vacuum aspiration (MVA) and misoprostol are the recommended treatment options for incomplete abortion in the first trimester, surgical management using sharp curettage is still one of the primary treatment methods in Malawi. Misoprostol and MVA are safer and cheaper, whilst sharp curettage has more risk of complications such as perforation and bleeding and requires general anesthesia and a clinician. Currently, efforts are being made to increase the use of misoprostol in the treatment of incomplete abortions in Malawi. To achieve successful implementation of misoprostol, health care providers’ perceptions on this matter are crucial.

**Methods:**

A qualitative approach was used to explore health care providers’ perceptions of misoprostol for the treatment of incomplete abortion using semi-structured in-depth interviews. Ten health care providers were interviewed at one urban public hospital. Each interview lasted 45 min on average. Health care providers of different cadres were interviewed in March and April 2021, nine months after taking part in a training intervention on the use of misoprostol. Interviews were recorded, transcribed verbatim and analyzed using ‘Systematic Text Condensation’.

**Results:**

The health care providers reported many advantages with the increased use of misoprostol, such as reduced workload, less hospitalization, fewer infections, and task-shifting. Availability of the drug and benefits for the patients were also highlighted as important. However, some challenges were revealed, such as deciding who was eligible for the drug and treatment failure. For these reasons, some health care providers still choose surgical treatment as their primary method.

**Conclusion:**

Findings in this study support the recommendation of increased use of misoprostol as a treatment for incomplete abortion in Malawi, as the health care providers interviewed see many advantages with the drug. To scale up its use, proper training and supervision are essential. A sustainable and predictable supply is needed to change clinical practice.

**Plain English Summary:**

Unsafe abortion is a major contributor to maternal mortality worldwide. Unsafe abortion is the termination of an unintended pregnancy by a person without the required skills or equipment, which might lead to serious complications. In Malawi, post-abortion complications are common, and the maternal mortality ratio is among the highest in the world. Retained products of conception, referred to as an incomplete abortion, are common after spontaneous miscarriages and unsafe induced abortions. There are several ways to treat incomplete abortion, and the drug misoprostol has been successful in the treatment of incomplete abortion in other low-income countries. This study explored perceptions among health care providers using misoprostol to treat incomplete abortions and whether the drug can be fully embraced by Malawian health care professionals. Health personnel at a Malawian hospital were interviewed individually regarding the use of the drug for treating incomplete abortions. This study revealed that health care providers interviewed are satisfied with the increased use of misoprostol. They highlighted several benefits, such as reduced workload and that it enabled task-shifting so that various hospital cadres could now treat patients with incomplete abortions. The health care workers also observed benefits for women treated with the drug compared to other treatments. The challenges mentioned were finding out who was eligible for the drug and drug failure. This study supports scaling up the use of misoprostol in the treatment of incomplete abortions in Malawi; the Ministry of Health and policymakers should support future interventions to increase its use.

## Background

Unsafe abortion is a major cause of maternal mortality, especially in countries with restrictive abortion laws [1]. Unsafe abortion is defined by the World Health Organization (WHO) as a procedure for terminating unintended pregnancies either by a person without the required skills and/or in an environment without the minimum medical standards [1]. In spite of improvements in the maternal mortality ratio (MMR) in Malawi, the MMR is still high at 439 per 100,000 live births, but not as high as some of the other countries in the Sub-Saharan region [2, 3]. Malawi has a restrictive abortion law, where termination of pregnancy is legal only to save a pregnant woman’s life [4]. Still, it is estimated that 140,000 induced abortions occur in Malawi every year, leading to many complications; these have a massive impact on the Malawian health system and contribute to the high maternal mortality in the country [2, 5, 6]. Some Malawian women with financial resources may have access to a safe abortion performed by skilled providers in private clinics [5]. Still, most of the induced abortions are conducted by traditional healers or women themselves using unsafe methods [7]. The most common complication after a miscarriage or an abortion is retained products of conception in the uterus which, if left untreated, can lead to bleeding, infections and death [8]. Even though complications can occur after safely induced abortions, that is rare, and most incomplete abortions occur after unsafe abortions or miscarriages [8, 9]. The high number of women needing treatment for complications after an abortion is a significant burden for the already impoverished health system [10, 11]. Liberalizing the restrictive abortion law has been discussed in the Malawian parliament for years, but so far, no change has been made [12]. Hence, efforts need to be made to increase the uptake of family planning and treating the complications after unsafe abortions.

Incomplete abortions can be treated with evacuation of the uterus, which can be done surgically, medically, or expectantly (waiting for spontaneous expulsion of the products) [1, 13]. WHO and The International Federation of Gynecology and Obstetrics (FIGO) recommend surgical treatment with manual vacuum aspiration (MVA) or medical treatment with misoprostol when the uterine size is 14 weeks or less [1, 14]. These two methods are cheaper, have less risk of complications, and are easier to use than sharp surgical curettage [1, 15]. In line with these recommendations, MVA is the recommended surgical treatment for incomplete abortions in Malawi [16]. However, studies have shown that sharp surgical curettage is used in most cases whilst misoprostol is rarely used [10, 17, 18]. Previous studies have shown that the use of misoprostol for treating incomplete abortion was as low as 1.3% in the southern part of Malawi [8]. This is in spite of the fact that misoprostol is available in public and private hospitals to treat postpartum hemorrhage and is accessible in local pharmacies on a physician’s prescription [19, 20]. It is possible that women can obtain misoprostol outside pharmacies, but no studies in Malawi have confirmed this yet. There are many reasons why sharp curettage is still the primary treatment method for incomplete abortions, even though it is against the guidelines. Malawian health care workers have reported obstacles in using MVA, such as broken equipment, lack of equipment, shortage of trained staff, and lack of time and support from the leadership [18]. Considering the challenges with MVA, increased use of misoprostol in post-abortion care (PAC) may be the way forward in Malawi and other low-income countries with restrictive abortion laws. A study in Malawi found that health personnel were aware of misoprostol as an option in treating incomplete abortions but were not using the method because they were unfamiliar with it [21]. Other reasons for the low use of misoprostol were fear of misuse or loss to follow-up [21, 22].

Several studies have recommended misoprostol in low-income countries, as its many practical advantages in low-resource settings might improve PAC [23–26]. Different barriers to implementing misoprostol have been identified, such as apprehensions related to its use at the provider and policy level, inadequate staffing, lack of knowledge of providers and end-users, and stock depletion [27]. Recently, an intervention study was done in five public hospitals in Malawi to increase the drug's use in PAC. Health care workers in intervention sites were trained on how to use misoprostol in PAC. Health care providers’ perceptions of the drug are crucial in the efforts being made to increase the use of misoprostol in the treatment of incomplete abortions in Malawi. The objective of this study was to explore health care providers' perceptions of the use of misoprostol in the treatment of incomplete abortions in Malawi.

## Methods

### Study design

A qualitative research design included face-to-face in-depth interviews using a semi-structured interview guide. The topic guide was developed based on a review of relevant literature and discussions among the authors and the interviewer.

### Study setting and intervention

The study was conducted at a public district hospital in Lilongwe. This district hospital covers an urban area in central Malawi. The facility was randomly selected as part of a larger intervention study on the use of misoprostol in the treatment of incomplete abortion. The hospital was selected for this qualitative study due to its accessible location in the urban area, and the number of health workers available for interviews. Health care providers at the hospital had participated in a training intervention in the use of misoprostol in the treatment of incomplete abortions as part of an intervention study. The training intervention took place in July 2020 and included a three-hour session on managing patients with incomplete abortions using misoprostol. During the training, health care workers were advised on patient assessment to determine eligibility for treatment. Women with incomplete abortion and a uterine size of less than 12 weeks’ gestation were given misoprostol 600 mcg orally or 400 mcg sublingually. Ibuprofen or paracetamol was used as pain relief. Women were kept in the hospital 4–6 h for observation. Those living within a short distance from the hospital were treated as outpatients. All women were offered family planning counseling and advised to return to the hospital if experiencing complications such as excessive bleeding or signs of infection. All women were advised to return for a follow-up visit one week later. The interviews with health care workers took place in March and April 2021.

### Study participants

All participants were health care workers involved in the management of incomplete abortion. Everyone interviewed, except one, had taken part in a training intervention in the use of misoprostol for incomplete abortion. The selection was made using purposive and convenience sampling. To create triangulation, various cadres, genders, and experience levels were recruited [28].

### Data collection

Interviews were conducted by a trained local data collector, familiar with the local setting and context, who was not a part of the research team. A topic guide covering areas such as advantages, disadvantages, consequences, and other personal experiences with the use of misoprostol was used. The interviewer was a Malawian registered nurse/midwife who had primarily worked in non-governmental organizations and was not in formal employment during the data collection period. Interviews were held in English as all participants were comfortable with the language. To ensure a comfortable setting, interviews were conducted individually in a private room at the facility. The interviews lasted around 45 min each. Interviews were recorded; to ensure anonymity, no names or personal information was included. Each participant gave written informed consent. Participation was voluntary, and participants were assured of privacy and confidentiality and were free to withdraw from the study at any point. No participants withdrew from the study.

### Data analysis

**Fig. 1:**
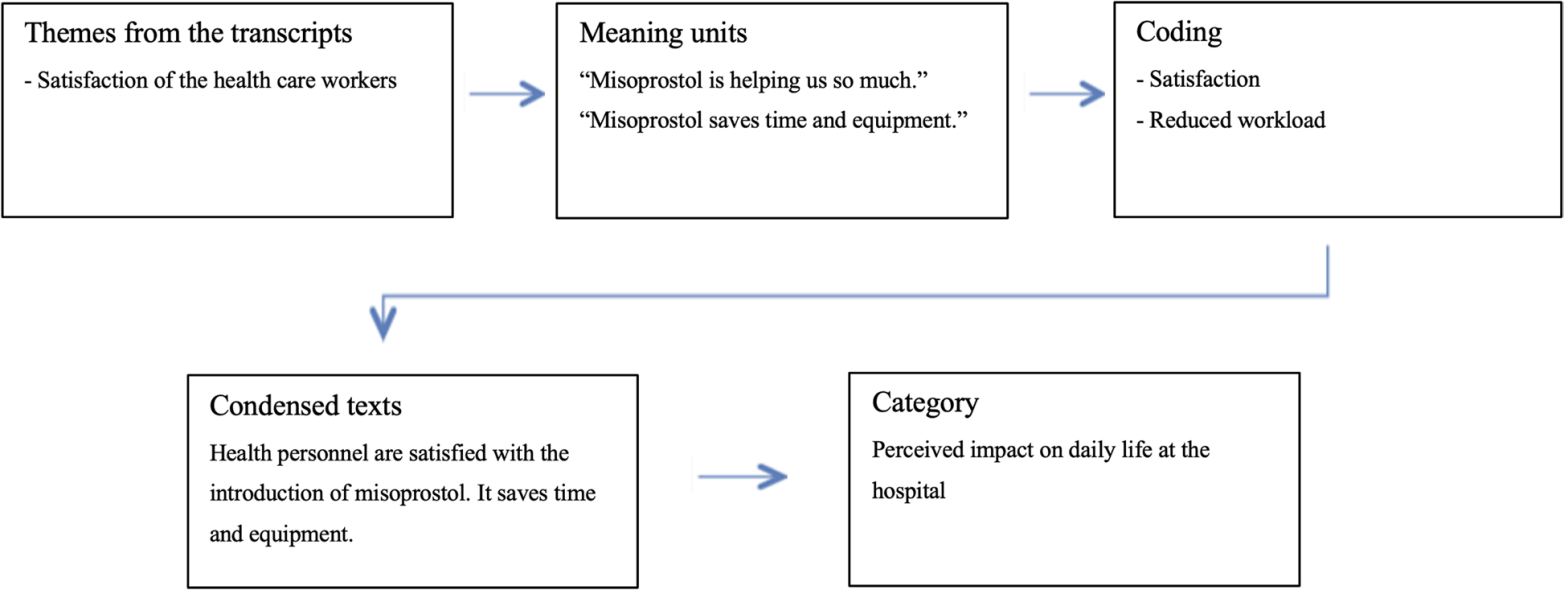
Example of the analytical process

The researchers analyzed the data in close collaboration and parallel to the data collection. The interviews were transcribed verbatim and were checked for clarity by the first authors. The interviews were analyzed using Systematic Text Condensation as described by Malterud [29]. The method consisted of four steps. First, the transcripts were read with an open mind several times to become familiar with the material and structure the impressions from chaos to themes. Next, the transcripts were reread to identify and sort out meaning units that might elucidate the study question. The preliminary themes were used to give similar statements and meanings a relevant code. The meaning units and codes were then condensed. The condensed texts were formed into analytical texts and given a final category. Results were illustrated by using anonymized quotes from the interviews. During the analytical process, some codes were merged, and some changed. NVivo software was used to organize the codes. After three interviews, a pre-analysis was done to get an overview of the data and ensure the data matched the aim of the study. To illustrate the process, an example of the analytical process is presented in Fig. 1.

## Results

A total of 10 healthcare workers were interviewed. The distribution of participating respondents, doctors, clinical officers, registered nurse/midwives, and nurse/midwife technicians is presented in Table 1.

**Table 1:** Characteristics of the informants

	Number	Gender (women /men)	Age (range)	Seniority^1^
Doctor	2	*Women* = *1, men* = *1*	28–32 years	7–8 years
Clinical officer^2^	2	*Women* = *0, men* = *2*	37–40 years	10*–*17 years
Nurse/midwife^3^	3	*Women* = *2, men* = *1*	25–29 years	2–2.5 years
Nurse/midwife technician^4^	3	*Women* = *2, men* = *1*	38–54 years	12- 26 years

^*1*^*Years of working experience in this cadre, *^*2*^*Formal Diploma in Clinical Medicine, *^*3*^*University Degree, *^*4*^*College Diploma in Nursing and Midwifery*

The main categories and sub-categories that emerged from the analysis are presented in Table 2.

**Table 2:** Categorization of the results

Categories	Sub-categories
Perceived impact on daily life at the hospital	Misoprostol is readily available Task-shifting Reduced workload
Observations of patients’ experiences	Fewer infections Time is not wasted No perforation and less pain
Health care providers’ personal experience	Effectiveness of misoprostol Challenges diagnosing the gestational age Follow-up

## Perceived impact on daily life at the hospital

### Misoprostol is readily available

In general, misoprostol was readily available at the hospital. However, some participants explained that misoprostol is sometimes out of stock, but only for a few days. They portrayed it as very challenging when they did not have access to the drug. One doctor explained that if they have little misoprostol, they prioritize patients with postpartum hemorrhage. Some participants pointed out that misoprostol had been less available in the past, but that the situation had improved. All participants agreed that the hospital management is supportive of the use of misoprostol as they try to make it available and encourage everyone to use it when indicated.

*Yes, by making sure that we have misoprostol throughout the year, I think they're [the management] being supportive. They took consideration that we really need that drug at this hospital. (...) We don't have shortages of misoprostol at this facility. It happens, but once in a blue moon.*

(Participant 6, nurse/midwife)

The participants stated that every health worker who needs misoprostol for treating patients could access the drug when it was available. However, it was important to document its use, because if personnel had free access to the drug they could take it and sell it in private hospitals and cause shortages in their own hospital.

### Task-shifting

The nurses observed less delay in treatment now because they did not have to wait for trained personnel to do MVA and curettage, as all nurses can provide complete treatment of incomplete abortion with misoprostol. The nurses were confident in treating patients with misoprostol if it was an incomplete abortion in the first trimester. Most participants thought misoprostol was easy to administer and monitor. The clinicians and doctors explained that they are very satisfied with the fact that they can delegate some of the management of incomplete abortions and stated that they do not have to be present during the treatment with misoprostol.

*Us clinicians, we are working doing the surgical management. But now, even nurses, once [you] give them the proper instructions, they will be able to give the misoprostol and monitor the patient. And if anything goes wrong, they will tell us.*

*(*Participant 5, doctor)

Most participants stated that they received no supervision from the management or leadership for the use of misoprostol. If there is a new employee, a fellow health care worker would explain how and when to use misoprostol. A nurse emphasized that she was happy with the supervision. A doctor thought most of the staff knew the dose and how to give misoprostol, and if people had not used it correctly, the management would have provided supervision. The nurse/midwife technicians would, however, liked to have more training. They wanted feedback on their work and to be updated on the indications, contraindications, and side effects regarding the use of misoprostol.

*At least, if someone could come around and see what we are doing. How we are managing our patients on misoprostol. (...) Would love to hear some compliment, yeah, maybe someone should say something: you are doing good, or you should improve on this.*

(Participant 10, nurse/midwife technician)

### Reduced workload

Several of the participants emphasized that misoprostol has lessened their workload as it takes a much shorter time to treat someone with misoprostol than with MVA or curettage. Participants explained that patients treated with misoprostol could be treated as outpatients, which reduced congestion in the hospital. Previously they had many patients in the ward as almost all patients receiving surgical treatment had to be admitted to the hospital. Hence, the health care providers were very content with misoprostol as many patients could travel home directly after the treatment. Additionally, one health care provider emphasized that they could now treat more patients than before.

*Well in the past we should do MVA for everybody, but I said... I mean, with less workload and you achieving the very same result that you used to achieve (…), that's something to be happy about, or satisfied.*

(Participant 5, doctor)

## Observations of patients’ experiences

### Fewer infections

Many participants highlighted that misoprostol reduces the risk of infection. They explained that although they try to be sterile when doing MVA, sometimes the equipment for the surgical procedures is not cleaned correctly, and they might introduce infection just by doing the procedure. Thus, they considered misoprostol to be safer than surgical procedures. One doctor explained that many patients had died of postabortal sepsis, and a crucial aspect of using misoprostol is reducing the risk of such complications. On the other hand, many health care providers were worried about patients with a pregnancy beyond the first trimester that have greater amounts of retained products. In these cases, one dose of misoprostol might not be efficient, and then the women might end up with sepsis due to treatment failure. Nevertheless, the providers stated that they had fewer admissions and fewer readmissions due to infection after using misoprostol, and that they were using fewer antibiotics as a consequence of this.

*It [misoprostol] helps and it is very important as it prevents infection, as (…) any invasive procedure, there is a risk of introducing some organisms into the patient’s body. So the use of misoprostol is safer than the use of MVA.*

(Participant 8, nurse/midwife)

### Time is not wasted

With misoprostol, there is no delay in the treatment, and therefore time is saved. This was highlighted as a benefit for the woman as she can go home to attend to her family or do other businesses instead of staying in the hospital.

*In misoprostol, the advantage is the patient doesn't stay long at the hospital. (...) After seeing the clinician, the patient returns the same day... While for MVA, it means the patient will be admitted.*

(Participant 3, clinical officer)

However, one doctor pointed out that if the woman has a lot of retained products, which usually happens with those beyond the first trimester, misoprostol does not work according to plan. These patients might end up staying longer at the hospital than if they had been treated with surgery.

### No perforation and less pain

Several participants mentioned their preference for misoprostol over MVA and curettage because of the risk of perforating the uterus when using invasive procedures. Misoprostol was therefore seen as safer for the patient, and the health care workers did not have to worry about such complications. The procedure of MVA was described as very painful for the woman, requiring the need for strong analgesics that are not always available. The patients might therefore only receive mild pain killers, making the procedure very painful. It was emphasized that the patient does not have to go through the whole process of anesthesia with misoprostol, and she does not have to experience post-operative pain.

*Yes [misoprostol] is beneficial to the patient because the procedure itself of MVA is very painful. And with the scarcity of the drug [strong pain killer], most of the time we don't use the required drug when doing MVA, like the Pethidine. No… We just give them sometimes Panadol, sometimes Diclofenac.*

(Participant 1, nurse/midwife)

Most patients with incomplete abortion have ongoing bleeding, and as misoprostol contracts the uterus, it helps control the bleeding. Patients also experienced side effects such as chills, fever, and diarrhea. However, in total, most participants stated that in their experience, misoprostol had more advantages than disadvantages for the patient.

## Health care providers’ personal experience

### Effectiveness of misoprostol

The participants were satisfied with the effectiveness of misoprostol. As long as the drug was prescribed according to the guidelines at the correct gestational age, it was perceived as effective. One of the health care workers clearly stated that misoprostol could be one of the most reliable ways for uterine evacuation at gestational ages below 12 weeks.

*I would say it's highly effective, yeah. Just that you have to use it to the right patient, yeah. If you use it to the right patient, it's effective.*

(Participant 5, doctor).

Even though they expressed satisfaction with the drug's effectiveness and that they were happier at work since the introduction of misoprostol, most participants were also concerned regarding challenges with its use. The primary concern was treatment failure and the woman returning with complications. Even though they thought the drug was successful in most cases, the health care providers were concerned that some of the participants returned with retained products. One of the nurses stated that even though only a small number of patients came back with retained products, this was still too many with complications. Many health care providers were particularly worried about patients returning with treatment failure and sepsis. Due to this, one clinical officer stated that he preferred MVA over misoprostol.

*But to the part of disadvantages, it’s what I’m saying, that some they don't produce the results which is needed. They come back and when the RPoCs* [retained products of conception] *are not yet finished, then we do manual vacuum.*

(Participant 2, nurse/midwife technician)

### Challenges diagnosing the gestational age

When choosing whether the patient should be treated with misoprostol, MVA, or evacuation with curettage, the participants explained that they had to estimate the gestational age of the pregnancy and the amount of retained products of conception. They mostly agreed that if the gestation age was less than twelve weeks, they would do MVA or give misoprostol. If there were small amounts of retained products, they would usually give misoprostol, and if there were more products, they would prefer MVA. Some participants explained that they would always send the patient for ultrasound scanning to determine the amount of retained products accurately. Others would only send the patient for a scan if they were unsure. If the gestation age was above 12 weeks and less than 27 weeks, curettage would be the preferred treatment. The participants explained that they could not give misoprostol when the gestational age was over 12 weeks as this could cause too many complications.

The common procedure for the pregnant women whose pregnancy had a gestational age of less than 9 weeks was to give misoprostol, and the health care workers were comfortable with this. However, several participants said it was difficult to determine the gestational age of a pregnancy above 8 or 9 weeks because it was hard to know if the pregnancy was in the first or second trimester. Hence, it was difficult to decide whether the woman was eligible for misoprostol. The health care providers agreed that history-taking was vital to set the correct gestational age, but this could be challenging. Some explained that history-taking depended on the health care worker to be very conversant and to be able to ask all the correct questions. Another challenge was that the patients were often unsure of their last menstrual period. There was also agreement that women lying about the time of their last menstrual period was a major problem. To detect this, the health worker had to be experienced in history-taking. The participants said that one of the reasons the woman would lie was because she knew that if she said she was less than 12 weeks pregnant, she would be prescribed misoprostol and could go home rather than having to stay in the hospital. One nurse said that if the drug did not work, she would not blame the health care worker who prescribed it or the drug itself, as she believed the main problem was women lying about the gestational age.

*So, … understanding the clear history, whether it’s indeed two or three months amenorrhea…, that's the tricky part.*

(Participant 7, clinical officer).

Using ultrasound scanning to determine the gestational age could be challenging. One doctor said that a problem was that sometimes the ultrasound might not be done instantly, and the woman had to wait. A nurse explained that not everyone trusted the expertise of the radiographer who did the scanning. She explained that in the beginning, the protocol was easy to follow: the patients with little retained products of conception would be given misoprostol. Later, some no longer trusted the results from the radiographers because they had experienced being given the wrong results. Some would, therefore, sometimes do an MVA even though the radiographer doing the scanning had reported “little” retained products of conception.

*Because sometimes you can say: we give Cytotec, and somebody would say: no, I'll do manual vacuum aspiration in this one, I don't trust the person who has done the scan (...). So usually MVAs are still happening.*

(Participant 6, nurse/midwife)

Another challenge with ultrasound was that the ultrasound scanning was not conducted at night. This meant the health workers had to trust their clinical examinations in order to decide whether to start managing the patient at once or keep the patient overnight with no treatment.

### Follow-up

The guidelines for the use of misoprostol at the hospital state that the woman should return to the hospital one week after the treatment to make sure all products are expelled. However, some health care providers stated they were not worried if the women did not come back for follow-up, as this probably indicated that the treatment had been successful. Hence, they were not concerned about the loss to follow-up and failed treatment causing infections and hemorrhaging.

*Well, looking at the nature of Malawians [laughing], I would say it's OK. Because knowing you are Malawian, if they feel better, if they feel fine, they are less likely to come to the hospital. So if somebody is not coming to the hospital, we assume everything is OK.*

(Participant 5, doctor)

Others expressed that they did not think the planned follow-up visits worked according to plan. They were not satisfied when the patients did not come back for a follow-up examination. One nurse/midwife technician said he liked the follow-up and wanted the women to come back, because it was good to know that he had successfully treated the patient. One nurse stated that better counseling on why the women should come back was needed and that if the women understood why it was essential to come back, they would.

*No, it doesn't work according to plan. It is really a few of them that comes back for the follow-up review. Most of the clients or patients that are being given misoprostol, they go home. They feel, or they see they have stopped bleeding, they don't even come for re-assessment.*

(Participant 7, clinical officer)

## Discussion

This study found that the health care providers experienced many advantages with the increased use of misoprostol. Even though most participants agreed that misoprostol was efficient, they also expressed concerns regarding ‘treatment failure’, which made some of them choose MVA over misoprostol. Earlier studies have shown that some health care workers doubt treatment with misoprostol and see more benefits with surgical treatment [17, 27]. However, the health care workers in this study underline that after gaining more experience in using misoprostol in treating incomplete abortions, they see it as an excellent option for the patients. This is in line with research in the field stating misoprostol is an effective treatment for incomplete abortion [13, 14, 23, 30].

Misoprostol has been found to be easy to use, and the health care providers at the hospital see this as a great advantage [23]. This is highlighted by Greenhalgh et al. as an important step for implementing innovations, who state that the successful assimilation of an innovation depends on the capacity and competence of the individual practitioner [31]. The situation at the gynecological ward was described as busy and overcrowded, and treatment with misoprostol was described as reducing the workload. Even though patients were not interviewed for this study, the health care providers expressed many advantages for the patients. The benefits mentioned were fewer infections, less use of antibiotics, less pain, less risk of perforation, and time-efficient treatment when it was used for the right patient. If the health care workers see advantages of the innovation and the current conditions are intolerable, they are more likely to adopt it [31, 32]. The likelihood that health care workers in this facility will continue to use misoprostol is therefore high.

The participants stated that misoprostol is usually readily available at the hospital. Consistent availability has likely contributed to the increased use of the drug at the hospital. This is further in line with Greenhalgh et al., who highlight stable funding as an important measure for an innovation to be implemented [31]. As part of the intervention trial, the drug was provided to the hospital at the onset of the intervention. The fact that the drug was still available one year after the intervention gives hope about sustained availability as the hospital showed commitment to maintaining the stock. Maintaining the drug at the hospital is in line with a study that found misoprostol available at many hospitals in Malawi [33]. Low availability and inconsistency in supplies of misoprostol are pointed out as reasons why implementation of misoprostol can be challenging in developing countries [27]. This was, however not found in this study.

Misoprostol ensures that nurses can provide the complete treatment of incomplete abortion. The participants in this study were very satisfied with task-shifting as they observed less delay in treatment. According to the guidelines, a commonly reported challenge for implementing PAC is staff shortage or inadequate staffing, and task-shifting could reduce this barrier [27, 34]. Task-shifting in the health sector in developing countries has been shown to be beneficial, safe, and effective; this aligns with the health care workers’ perceptions in this study [35–38]. Lack of supervision and training as a barrier to implementing misoprostol is rarely found. The importance of training and supervision should, however, not be forgotten [13, 27, 31, 34, 39, 40]. Most of the nurse/midwife technicians in this study would prefer more proper training, which has also previously been found in Malawi [21]. Training can be done frequently at little cost. Lack of support from the leadership and key stakeholders has been indicated as one of the most significant barriers to implementing misoprostol for PAC [27]. However, this reluctance does not seem to be present among the management at the studied Malawian hospital. The implementation might therefore run smoother there than in other developing countries.

In this study, health workers mentioned treatment failure as a disadvantage of misoprostol. A possible explanation for the treatment failure could be substandard misoprostol tablets, as found by Hagen et al. [33]. Moreover, the health care workers mentioned that if patients with a lot of retained products or higher gestational age than expected received misoprostol, they could develop sepsis because of treatment failure. Appropriate screening to identify patients who meet the criteria for treatment with misoprostol is, therefore, a crucial part of successfully implementing the drug. Ultrasound can diagnose the gestational age, ensure the uterus is emptied after treatment (follow-up), and determine the amount of retained products [13, 41–43]. Participants in this study mentioned several challenges in using ultrasound to determine gestational age: Patients had to wait for trained personnel to do the scanning and the scanning was not available at nighttime. Additionally, not all health care workers trusted the expertise of the radiographers, which might be due to some radiographers’ lack of experience. When a higher gestational age is suspected, women could be observed at the hospital and potentially taken to surgery if treatment with misoprostol was unsuccessful. Priming the cervix with misoprostol before surgical procedures is common when treating incomplete and missed abortions in high-income countries and does not harm patients who end up needing surgical evacuation of the uterus [44–46].

Local guidelines state that women treated with misoprostol should return for follow-up after one week. These guidelines are based on the WHO guidelines from 2014, which recommended follow-up after treatment with misoprostol [30]. According to the new WHO guidelines from 2022, a follow-up is not routinely needed after medical abortion [1]. The local guidelines at the hospital might change according to the new guidelines from WHO and after health care workers in Malawi become more familiar with the use of misoprostol. The informants in this study state that many patients did not show up for their follow-up appointment. Despite this, many of them said they still thought the follow-up worked because if the woman did not return, it meant the treatment was successful, and she was not experiencing any complications. Loss to follow-up after treatment with misoprostol is a known problem, and lack of money and transportation have been mentioned as barriers for the woman to return for her follow-up appointment [26, 47]. Alternatives to routine in-person follow-up have been researched, with urine pregnancy testing, self-assessment, and telephone consultation being shown to be potential solutions to this issue [42, 43]. The lack of ultrasound in low-resource settings should, therefore, not exclude the introduction of misoprostol, as there are still many benefits from using this drug [39]. Furthermore, the WHO states that using ultrasound for diagnosing incomplete abortion might lead to unnecessary surgical interventions [13].

Complications from unsafe abortions account for many admissions to the gynecological departments in developing countries [48]. The maternal mortality ratio is high in Sub-Saharan Africa, and initiatives to reduce maternal deaths are important [3]. Studies have shown that unsafe abortions and related deaths could decrease when abortion laws are more liberalized [8, 49–51]. There are discussions in Malawi about changing the abortion law. However, no changes have been implemented at this point [12]. Even if the abortion law of Malawi should be liberalized, unsafe abortions will still occur. Hence a good and available PAC will always be necessary. The implementation of misoprostol for treating incomplete abortion is, therefore, an important matter to study as part of the effort to reduce maternal mortality and morbidity in settings like Malawi [52].

## Strengths and limitations of the study

This is the first study conducted on health workers’ perceptions of using misoprostol in Malawi. A strength of this study was the collaboration between Norwegian and Malawian researchers, which helped gain a deeper understanding of the data. A heterogeneous group of health professionals was selected to achieve a broad range of perspectives. Open questions were used in the interviews, and the participants were encouraged to speak freely and to be honest and were assured that their answers would have no consequences on their work. There is, however, always a risk that participants answer what they think the researcher wants to hear or what is expected of them. However, this risk was reduced as the data collector was a Malawian nurse/midwife who had never met the participants before.

The first authors analyzed the transcripts, since multiple researchers can strengthen the study by supplementing and contesting each other's statements [53]. Potential preconceptions were discussed before, during, and after the data collection and analysis. During the process, the Norwegian authors were aware of how their background and preconceptions about Malawi could impact the analysis [53].

This small-scale study involves only health care workers at a busy district hospital in Malawi. Thus, transferability to other settings may be limited. As health centers in rural areas often are the first contact for women seeking PAC, further research involving these health centers would be beneficial.

## Conclusion

Increasing the use of misoprostol in the treatment of incomplete abortion has many advantages, especially in developing countries. The findings in this study support the increased use of misoprostol as a treatment for incomplete abortion in Malawi, as the health care providers interviewed see many advantages with the drug. To scale up its use, proper training and supervision are essential to assist health care workers to screen and identify patients to be treated with misoprostol. A sustainable and predictable supply of misoprostol is needed to change clinical practice. This study supports the introduction of misoprostol in the treatment of incomplete abortions in Malawi, and the Ministry of Health and policymakers should support future interventions to increase its use.

## Data Availability

The transcribed interviews are available from the corresponding author upon receiving a reasonable request.

## Abbreviations

WHO: World Health Organization
ACOG: American College of Obstetricians and Gynecologists
FIGO: International Federation of Gynecology and Obstetrics
MVA: Manual Vacuum Aspiration
PAC: Post-abortion care
MMR: Maternal mortality ratio
RPOC: Retained Products of Conception
COMREC: College of Medicine Research Ethics Committee
REK: Regional Committee for Medical and Health Research Ethics
